# KEA-1010, a ketamine ester analogue, retains analgesic and sedative potency but is devoid of Psychomimetic effects

**DOI:** 10.1186/s40360-019-0374-y

**Published:** 2019-12-19

**Authors:** Martyn Harvey, Jamie Sleigh, Logan Voss, Mike Bickerdike, Ivaylo Dimitrov, William Denny

**Affiliations:** 1 Emergency Department, Waikato Hospital, Pembroke St, Hamilton, 3240 New Zealand; 2 Anesthesia Department, Waikato Hospital, Pembroke St, Hamilton, 3240 New Zealand; 3 Kea Therapeutics Ltd, Lumley Centre, 88 Shortland Street, Auckland, New Zealand; 4 Auckland Cancer Society Research Centre, University of Auckland, Park Rd, Auckland, New Zealand

**Keywords:** Ketamine, Analogue, Analgesia, Pain

## Abstract

**Background:**

Ketamine, a widely used anaesthetic and analgesic agent, is known to improve the analgesic efficacy of opioids and to attenuate central sensitisation and opioid-induced hyperalgesia. Clinical use is, however, curtailed by unwanted psychomimetic effects thought to be mediated by *N*-methyl-D-aspartate (NMDA) receptor antagonism. KEA-1010, a ketamine ester-analogue designed for rapid offset of hypnosis through hydrolysis mediated break-down, has been shown to result in short duration sedation yet prolonged attenuation of nociceptive responses in animal models. Here we report on behavioural effects following KEA-1010 administration to rodents.

**Methods:**

KEA-1010 was compared with racemic ketamine in its ability to produce loss of righting reflex following intravenous injection in rats. Analgesic activity was assessed in thermal tail flick latency (TFL) and paw incision models when injected acutely and when co-administered with fentanyl. Tail flick analgesic assessment was further undertaken in morphine tolerant rats. Behavioural aberration was assessed following intravenous injection in rats undergoing TFL assessment and in auditory pre-pulse inhibition models.

**Results:**

KEA-1010 demonstrated an ED_50_ similar to ketamine for loss of righting reflex following bolus intravenous injection (KEA-1010 11.4 mg/kg [95% CI 10.6 to 12.3]; ketamine (racemic) 9.6 mg/kg [95% CI 8.5–10.9]). Duration of hypnosis was four-fold shorter in KEA-1010 treated animals. KEA-1010 prolonged thermal tail flick responses comparably with ketamine when administered de novo, and augmented morphine-induced prolongation of tail flick when administered acutely. The analgesic effect of KEA-1010 on thermal tail flick was preserved in opioid tolerant rats. KEA-1010 resulted in increased paw-withdrawal thresholds in a rat paw incision model, similar in magnitude yet more persistent than that seen with fentanyl injection, and additive when co-administered with fentanyl. In contrast to ketamine, behavioural aberration following KEA-1010 injection was largely absent and no pre-pulse inhibition to acoustic startle was observed following KEA-1010 administration in rats.

**Conclusions:**

KEA-1010 provides antinociceptive efficacy in acute thermal and mechanical pain models that augments standard opioid analgesia and is preserved in opioid tolerant rodents. The NMDA channel affinity and psychomimetic signature of the parent compound ketamine is largely absent for KEA-1010.

## Background

Managing severe pain in the emergency room, and the peri-operative period, is complex. While opioid analgesics remain the mainstay of treatment for severe pain, increasing numbers of patients with chronic pain states and opioid dependence can challenge the ability of traditional analgesic regimes to provide adequate pain control [1–4]. Opioid-induced hyperalgesia and analgesic tolerance are frequent drivers of diminished pain control and dose escalations in the hospital setting [5, 6].

**Fig. 1:**
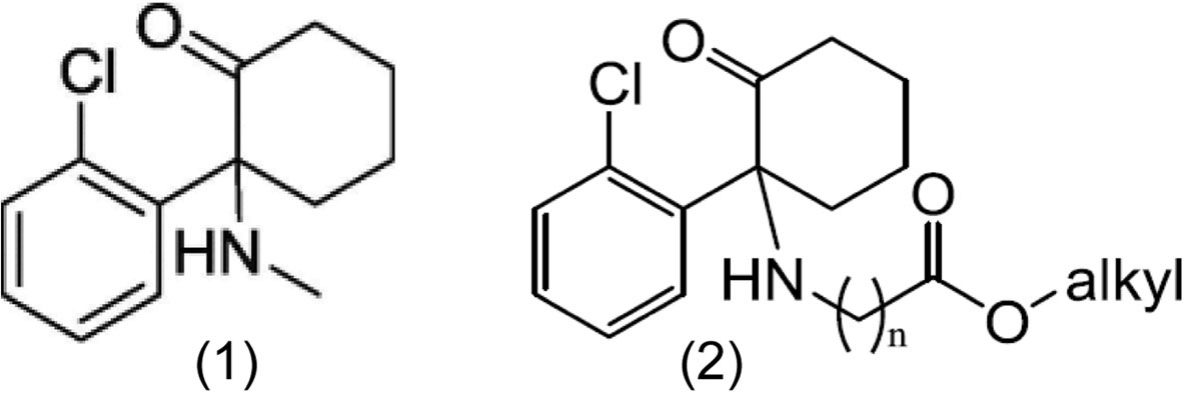
Ketamine structure (1); N-Aliphatic ester-analogues of ketamine – generic structure (2)

Ketamine (Fig. 1) [1] is most widely recognised as a non-competitive antagonist at the phencyclidine site of the N-methyl-D-aspartate receptor (NMDAR) in both the central and peripheral nervous system [7–9]. Administration at hypnotic doses produces dissociative anaesthesia while sub-hypnotic dosing has been shown to attenuate central sensitization and hyperalgesia [10, 11], and to reduce overall opioid consumption in the immediate post-operative period [12, 13]. Antinociceptive effects following de novo administration have been documented in humans [14–18] and rodents [19–21]. Attendant psychomimetic effects, however, limits more widespread clinical application [13, 22, 23].

Engineered N-Aliphatic ester-analogues of ketamine Fig. 1 [2] which were developed to undergo tissue-based metabolism to inactive carboxylic acid by-products, have demonstrated rapid offset hypnosis in animal models [24–26]. Prior work in rodents has been undertaken to determine optimum side chain length and functional groups to maximise therapeutic sedative and antinociceptive actions of these novel agents [27]. Use of such analogues of ketamine has enabled titration of dose to hypnotic effect, while maintaining expeditious arousal following cessation of infusion in animal models [25]. One such analogue, KEA-1010, has demonstrated persisting antinociceptive responses to thermal and mechanical nociceptive stimuli, that far out-last any hypnotic action. Effects were observed in the absence of any significant ketamine-like aberration in behaviour. These data suggest a potential role for novel agents such as KEA-1010 in clinical management of severe pain, wherein a ketamine-based drug with antinociceptive action but *lacking* psychomimesis would prove beneficial.

**Fig. 2:**
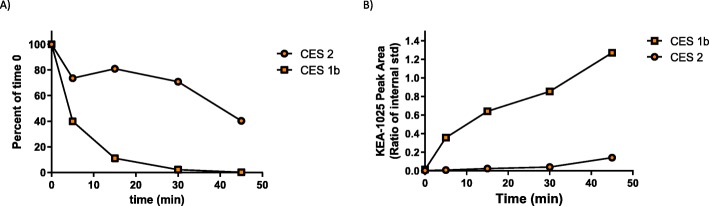
KEA-1010 hydrolysis is more rapid when exposed to CES1b expressing Supersomes in vitro. KEA-1010 concentration (as percent of time zero) following incubation with CES1b and CES2 expressing Supersomes in vitro (**a**), and: accumulation of the compound’s carboxylic acid metabolite (KEA-1025) in the same incubations (**b**), confirming that rapid degradation of the parent ester is largely by CES-1 type enzymes, found predominantly in the liver

In the present series of investigations KEA-1010 was tested in rodent models to determine sedative thresholds, responses to thermal and mechanical nociceptive stimuli, efficacy in combination with opioid analgesic medications, and for adverse behavioural changes following administration. Our results reveal rapid offset of hypnosis yet a prolonged antinociceptive action to painful thermal and mechanical insult which proved additive with traditional opioid analgesics. Effectiveness in opioid tolerance was observed. Beneficial effects were largely devoid of adverse behavioural effects seen with ketamine.

## Methods

### Animals

All animal experiments were conducted in accord with recommendations of the National Institutes of Health Guide for the Care and Use of Laboratory Animals. Determination of ED_50_ and nociceptive testing was undertaken at the Ruakura Research Centre, Hamilton, New Zealand, using experimental protocols reviewed and approved by the Ruakura Animal Ethics Committee. Pre-pulse inhibition experiments were undertaken by the contract research company RenaSci Ltd., Nottingham, UK and ethical approval for the study was approved by the Nottingham Animal Ethics committee. For all experiments adult (200–350 g) Sprague-Dawley rats (163 female for ED_50_ determination and nociceptive testing [sourced from Ruakura rodent breeding colony], 36 male for pre-pulse inhibition [purchased from Charles River UK]) were studied. Rats were housed in groups of 4 under a 12 h/12 h light/dark cycle (lights on at 07.00 h), at an ambient temperature of 21 ± 2 °C and 55 ± 20% humidity. Food (standard pelleted diet) and water were available ad libitum. Animals were allowed to acclimatise to these conditions for approximately 1 week prior to use and underwent a handling protocol beforehand so that they are used to manual handling. Animal allocation was via computerised random number generation. All experiments were performed in the light phase of the light/dark cycle. At the completion of experiments animals were killed via carbon dioxide narcosis and cervical dislocation. All investigators were blinded to study drug administration.

### Drugs and reagents

The ketamine analogue KEA-1010, and its primary carboxylic acid metabolite (KEA-1025) when appropriate, were the study drugs of key interest. In behavioural experiments an additional ketamine analogue (KEA-1037), notable for significant adverse psychomimetic effects was employed. KEA-1010, KEA-1025, and KEA-1037 were synthesised from norketamine by the Auckland Cancer Research Society laboratory, Auckland, New Zealand. Final product was analysed by reverse-phase HPLC (High performance liquid chromatography; Alltima C18 5 μm column, 150 × 3.2 mm; Alltech Associated, Inc., Deerfield, IL) using an Agilent 1100 LC equipped with a diode array detector. The mobile phase was 80% CH_3_CN/20% H_2_O (v/v) in 45 mM HCO_2_NH_4_ at pH 3.5 and 0.5 mL/min. The purity was determined by monitoring at 272 nm and was ≥95%. Commercially available racemic ketamine (Hospira Australia Ltd., VIC, Australia), fentanyl citrate (Mercury Pharma, Sydney, Australia), and morphine sulphate 30 mg/mL (Pfizer, Auckland, New Zealand) were purchased from credentialed suppliers. All drugs were diluted in 0.9% saline prior to administration.

### Confirmation of rapid esterase degradation

We utilised two different carboxylesterase isoforms (CES1b and CES2) to investigate the direct action of individual enzymes on KEA-1010 metabolism. CES1 is abundantly expressed in the liver and adipocyte, with lesser amounts in the kidney, monocytes, lung, intestine, testis, heart, and macrophages. In contrast CES2, while additionally expressed peripherally, is also expressed in the brain [28]. In vitro pharmacokinetic studies of KEA-1010 were conducted in specific supersomes which recombinantly expressed CES1b or CES2 enzymes in vesicles, to confirm esterase degradation characteristics. In vitro incubations were prepared with Supersomes in 0.1 M phosphate buffer pH 7.4 at 1 mg/kg protein concentration. Reaction mixtures were pre-incubated at 37 °C with shaking for 10 min using an Eppendorf ThermoMixer Comfort (Stevenage, UK). Incubations were initiated by the addition of KEA-1010 (final concentration 1 μM, 0.25% DMSO) and baseline samples were taken immediately and quenched in ice cold acetonitrile containing formic acid 1%. Incubations were sampled at 5, 15, 30, and 45 min. Supernatants were diluted 1:1 with 0.55 μM metoprolol (internal standard) in water for liquid chromatography-tandem mass spectrometry (LC-MS/MS) analysis of KEA-1010 and its primary carboxylic acid metabolite (KEA-1025). Five replica trials were undertaken.

### NMDA receptor binding affinity

Determination of NMDA inhibition was carried out commercial provider (Eurofins Panlabs Taiwan, Ltd. Pharmacology Laboratories) according to reported procedure [29]. MK-801 {[5*R*,10*S*]-[+]-5-methyl-10,11-dihydro-5*H*-dibenzo [*a*,*d*]cyclohepten-5,10-imine}, Ketamine and KEA-1010 were tested for competitive binding against the radioligand 5 nM [^3^H]-MK-801 in wistar rat brain preparations, incubated in 5 mM Tris-HCL at pH 7.4 for 3 h at 25 °C. Radioligand displacement was used to determine ligand binding affinity to calculate IC_50._

### In vitro ligand-receptor binding evaluation

To explore the fundamental molecular mechanism (s) of study compounds a suite of receptor, ion channel, and other protein binding profiles was sourced from a commercial provider (Eurofins Panlabs Ltd., Taipei, Taiwan). Duplicate studies were undertaken at concentrations 10 μM, 1 μM, 0.1 μM, and 10 nM, for the compounds: ketamine, KEA-1010, and the carboxylic acid metabolite of KEA-1010 (KEA-1025). Detailed explanations of the methodology employed in potential-target profiling are reported elsewhere, and can be found at http://www.eurofinspanlabs.com/Panlabs using the number listed in parentheses after each assay. Potential-target profiling was performed at the following targets: calcium channel L-type, benzothiazepine (214510); opiate δ1, OP1 DOP (260130); Opiate μ OP3 MOP (260410); norepinephrine uptake (302000); and hyperpolarization-activated cyclic nucleotide-gated ion channel, HCN1 (952727). In receptor binding studies performed at Eurofin Panlabs, > 50% inhibition of binding was deemed significant. Reference standards were run as an integral part of each assay to ensure the validity of the results obtained.

### Determining ED_50_ for loss of righting reflex (LORR)

Hypnotic thresholds for KEA-1010 and ketamine were determined following bolus intravenous administration. Loss of righting reflex (LORR) was primarily used to assess hypnotic effect. Following cannulation of the marginal tail vein rats were injected over a 30 s period with KEA-1010 or ketamine in 1 mL 0.9% saline. Initial doses were at 3 and 30 mg/kg with subsequent dosing determined in an up-and-down fashion. Righting reflex was judged absent when a rat failed to right from a position of dorsal habitus to a position of sternal habitus after three attempts performed in rapid succession. Duration of loss of righting reflex was deemed time from onset of LORR to time to return of righting reflex (RORR), equal to spontaneous righting from a position of dorsal habitus to a habitus of sternal habitus.

### Nociceptive testing

#### Thermal sensitivity (tail Flick) testing

A tail flick analgesia meter (Colombus Instruments, Colombus, Ohio) was used to determine thermal pain sensitivity following KEA-1010 administration. Radiant heat was applied using a shutter-controlled lamp as a heat source focussed on three different points on the volar aspect of the middle third of the tail. The intensity of the beam was set at a level producing basal latency times between 2 and 4 s prior to drug administration. To prevent thermal tissue injury the cut-off time as set at 10 s. A digital response time indicator with a resolution of 0.01 s measured the time from initiation of stimulus until tail withdrawal (the flick; TFL).

Rat responses to thermal tail insult were explored following bolus intravenous injection, and 10 minute intravenous infusion employing regimens demonstrated to produce overt hypnosis (defined by maintained loss of righting reflex), and at sub-hypnotic dosing (no loss of righting reflex). Ketamine was employed as active control. Our test paradigm was as follows:

- *Hypnotic injection*: KEA-1010 at 11 mg/kg over 15 s; ketamine 10 mg/kg over 15 s.
- *Sub-hypnotic injection*: KEA-1010 at 5.5 mg/kg over 15 s; ketamine 5 mg/kg over 15 s.
- *Hypnotic infusion*: KEA-1010 at 20 mg/kg/min for 1 minute followed by 4 mg/kg/min for 9 min (total 56 mg/kg); ketamine 10 mg/kg/min for 1 minute followed by 2 mg/kg/min for 9 minutes (total 28 mg/kg).
- *Sub-hypnotic infusion*: KEA-1010 at 4 mg/kg/min for 10 minutes (total 40 mg/kg); ketamine at 2 mg/kg/min for 10 minutes (total 20 mg/kg).

Recordings of TFL were undertaken for 60 min. Duration of loss of righting, when present, was recorded.

#### Mechanical sensitivity (Von Frey filament) testing

To assess mechanical sensitivity, the withdrawal threshold to von Frey filament stimulation after hind paw incision and suture was undertaken. Following acclimatisation in clear plastic cages with mesh flooring for 60 min rats underwent Isoflurane (Piramal Healthcare, Auckland, New Zealand; SomnoSuite small animal anaesthetic system, Kent Scientific, Torrington, CT, USA) anaesthesia via perspex chamber (4%), and subsequent nose cone (2%). During 10 minute anaesthesia KEA-1010 at 20 mg/kg, fentanyl at 10 μg/kg, KEA-1010 at 20 mg/kg *and* fentanyl at 10 μg/kg, or 2 mL 0.9% saline was infused via tail vein according to prior randomisation.

While on a warming plate (38 °C) the plantar aspect of the right hindpaw was prepared in a sterile manner with 1% chlorhexidine solution and draped. A 1 cm incision was made on the plantar aspect of the foot starting 5 mm from the heel and extending toward the toes with a No. 15 blade. The incision extended through the skin and fascia to expose the plantaris muscle which was then elevated and longitudinally incised – leaving both the origin and insertion of the muscle intact. After haemostasis with gentle pressure the skin was closed with two mattress sutures of 5.0 ethylon on a curved needle, and animals returned to individual enclosures.

The von Frey filaments (Aesthesiometer, Somedic, Sweden) were used at 5 min, 10 min, 20 min, 30 min, 60 min, 2 h, 4 h, and 6 h following paw incision to evaluate the magnitude of mechanical allodynia. For this method filaments of varying size (8–18, corresponding to [in grams] 0.92–70) were applied perpendicular to the plantar surface adjacent to the wound until the filament flexed and held in place for 5 seconds. The 50% withdrawal threshold was determined using the up-and-down method as previously described [30]. A positive response was deemed brisk paw withdrawal, shaking, or licking of the hind paw. Weight bearing status at each time point was scored according to a binary scale indicating the position the hind foot was found during the majority of a one-minute observation period immediately prior to von Frey assessment.

#### Induction of opioid tolerance and thermal sensitivity testing

Opioid tolerance was induced with continuous administration of morphine via implanted osmotic minipumps (Alzet 2ML1, Durect, Cupertino, CA, USA) in line with the work of Lilius et al. [31]. The pumps were prefilled with 2 mL morphine at 25 mg/mL in isotonic saline solution to deliver a constant dose of 6 mg of morphine daily. In control animals 0.9% saline solution was used as vehicle.

Pumps were implanted under brief general anaesthesia (identical to that of paw incision). Following clipping and sterile preparation a 2 cm interscapular incision and blunt dissection permitted pump localisation on the back. Wounds were closed with surgical clips and rats returned to solitary enclosures to recover before returning to group cages.

Thermal tail flick latency was documented at baseline (pre-implantation of minipumps), and daily thereafter to document development of opioid tolerance. The effect of KEA 1010 and ketamine on augmentation of morphine-induced analgesia was documented on day one following intravenous administration of KEA-1010 (12 mg/kg over 30 s), ketamine (12 mg/kg over 30 s), and 0.9% saline control (1 mL over 30 s). Righting status and tail flick latency was undertaken at 10 min intervals to 60 min following administration of intravenous treatments. Behavioural dysfunction scoring was undertaken (Table 1) to 60 min. On day six, after confirming the development of opioid tolerance with TFL testing, intravenous treatments were repeated and righting status, TFL, and behavioural dysfunction scoring again recorded to 60 min.

**Table 1:** Behavioural dysfunction scoring

1) General	Exopthalmos
	Increased respiration
	Decreased respiration
2) Motor	Subdued
	Increased/decreased body tone
	Increased/decreased locomotor activity
3) Reactivity	Increased irritability on gentle handling
	Decreased irritability on gentle handling
	Rearing
4) Stereotypical behaviours	Head weaving
	Ataxia/decreased co-ordination
	Splayed hind legs

A score of 1 or zero is allowed for each of the four categories permitting a maximum score of four

(Modified from Koros et al. 2007 [53])

### Pre-pulse inhibition (PPI) testing

Eight startle chambers (TSE Systems GmbH, Bad Homburg, Germany) were used to measure startle reactivity in rats. The startle test chambers consist of small Plexiglas and wire mesh cages on top of a vibration-sensitive platform in a sound-attenuated, ventilated enclosure. A high-precision sensor, integrated into the measuring platform, detects movement which is recorded by computer. Two high-frequency loudspeakers inside the chamber produce all the audio stimuli. A dynamic calibration system was employed to ensure comparable stabilimeter sensitivity across test chambers, and sound levels were measured using the dB (A) scale. Individual animals were always evaluated in the same chamber.

KEA-1010 was compared with saline control and ketamine during test sessions. Another Kea Therapeutics’ ketamine analogue, KEA-1037, was included as a further positive control as preliminary studies demonstrated it induced psychotomimetic-like stereotypies (data not shown). Animals were placed in the testing chambers 5 min after dosing with saline 0.9% 1 mL IV; ketamine 10 mg/kg IV, KEA-1010 11 mg/kg IV, or KEA-1037 at 10 mg/kg. Sessions began with 5 min acclimation to white background noise [62 dB] which will be maintained through the whole session. Rats were then exposed to 2 blocks of trials. The first 10 trials consisted of 10 pulse trials (i.e. no pre-pulse) to habituate the animals to the startle. Subsequently, rats received 28 trials consisting of pulse alone trials, trials of pulse preceded by a pre-pulse (either 70, 73, 76 dB), and trials of the pre-pulse only (*n* = 4). These were presented in a random order. The inter-trial interval was 20s and the startle pulse was 120 dB for 40 ms duration. Pre-pulses were presented 100 ms (onset to onset) before the pulse and duration was 20 ms. In total animals received 38 trials per session. Animals were removed from startle chambers to undergo behavioural testing at 23 min, before returning for repeated startle assessment at 30 min.

### Statistical analysis

Statistical analyses were conducted using GraphPad Prism 5.0 (GraphPad Software Inc., La Jolla, CA). Probit analysis was used to fit dose-response curves and to estimate ED50 for each study agent. Behavioural data were analysed using repeated measures (RM) analysis of variance (ANOVA) models with Bonferroni post-testing when significance was achieved. Clinical observations were analysed by exact Wilcoxon rank sum test. Categorical data were analysed using Fishers exact testing. IC50 values were determined by a non-linear, least squares regression analysis. The significance level was set at *P* < 0.05 in all statistical analyses.

## Results

### In vitro confirmation of rapid esterase breakdown

In vitro half-life (t_1/2_) for KEA-1010 was 5.41 min when incubated with CES1-expressing Supersomes, and 42.6 min when incubated with CES2-expressing Supersomes (Fig. 2a). Accumulation of the primary carboxylic acid metabolite of KEA-1010 (i.e. KEA-1025) was correspondingly greater in CES1 preparations (Fig. 2b).

### Low affinity NMDA receptor binding

NMDA receptor binding affinities for MK-801, ketamine, KEA-1010, and KEA-1025 are presented in Table 2. Notably the IC_50_ for KEA-1010 (> 100 μM) was significantly lesser than ketamine (< 1 μM).

**Table 2:** NMDA receptor binding affinity

Agent	IC_50_
MK-801	7.13 nM
Ketamine	0.7 μM
KEA-1010	134 μM
KEA-1025	313 μM
KEA-1037	2.7 μM

### Receptor binding profiles

Binding profiles for the receptor, ion channel, and other protein targets investigated is presented in Table 3. KEA-1010 is notable for is absence of significant inhibition at any receptor target, and weak inhibition of the HCN1 potassium channel in these assays.

**Table 3:** Molecular target binding profiles

Molecular Target (Assay Type)	Ketamine	KEA-1010	KEA-1025
	IC_50_ [Percent Inhibition at 10 μM]		
Ca^2+^ Channel L-Type (Radioligand Binding)	> 10 μM [24%]	> 10 μM [−]	> 10 μM [−]
GABA_A_ Receptor (Radioligand Binding)	> 10 μM [−]	> 10 μM [−]	> 10 μM [−]
δ_1_ Opiate Receptor (Radioligand Binding)	> 10 μM [−]	> 10 μM [−]	> 10 μM [−]
μ Opiate Receptor (Radioligand Binding)	> 10 μM [−]	> 10 μM [−]	> 10 μM [−]
Norepinephrine Uptake Site (Functional Uptake Assay)	> 10 μM [23%]	> 10 μM [−]	> 10 μM [−]
HCN1 K^+^ Channel (Patch-Clamp Functional Assay)	> 100 μM [15%]	> 100 μM [20%]	> 100 μM [−]

Percent Inhibition at 10 μM only quoted where inhibition > 15%

Percent Inhibition in patch-clamp assay quoted at 100 μM, not 10 μM, as that was top dose employed

### Sedative characteristics of KEA-1010

**Fig. 3:**
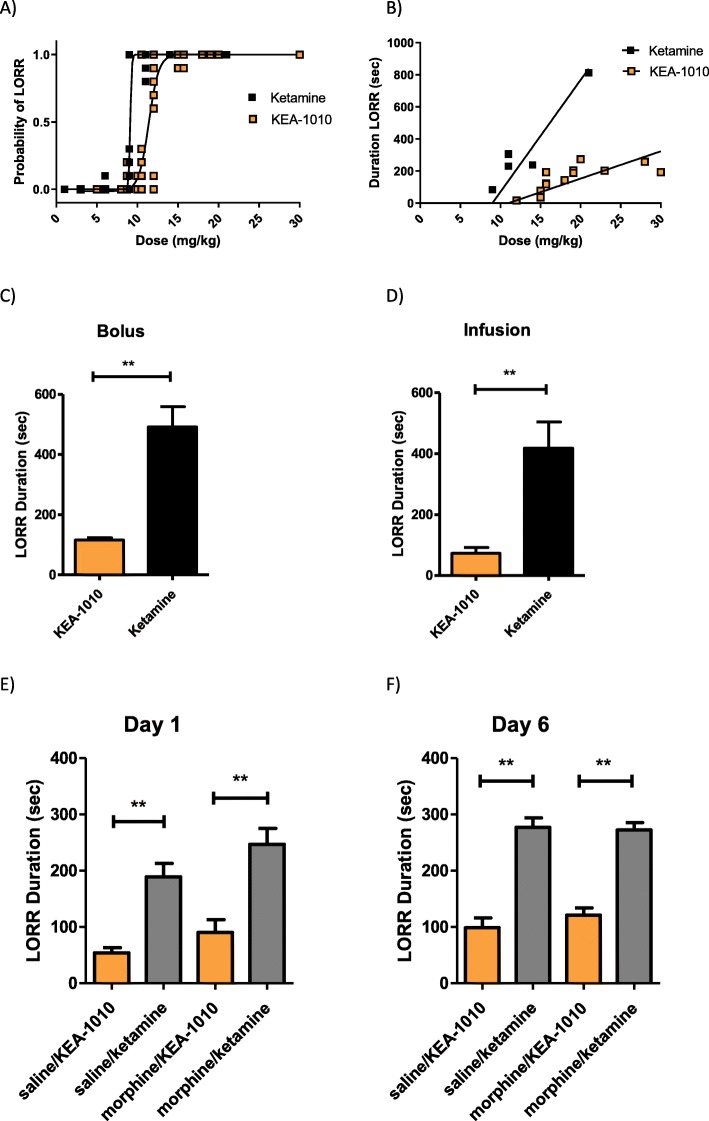
Duration of KEA-1010 induced loss of righting reflex is shorter than ketamine. Probability of loss of righting reflex (LORR) following intravenous injection of KEA-1010 and ketamine over 30 s (**a**). ED_50_ for LORR KEA-1010 is 11.4 mg/kg (95% CI 10.6 to 12.3); ED_50_ for LORR ketamine (racemic) is 9.6 mg/kg (95% CI 8.5–10.9). Duration of LORR following intravenous injection of KEA-1010 and ketamine over 30 s (**b**). Slope of LORR duration is KEA 1010 17.0 s/mg/kg (95% CI 12.6 to 21.4); ketamine 69.8 s/mg/kg (95% CI 45.1 to 94.5). Duration of LORR following KEA-1010 and ketamine via intravenous bolus (**c**) and 10 minute infusion (**d**) at hypnotic dosing in rats. Duration of LORR on day 1 (**e**) and day 6 (**f**) following intravenous bolus injection of KEA-1010 and ketamine in rats receiving either saline, or morphine, via subcutaneous minipump. Data: mean (SEM). **P < 0.01 (Students T testing). *N* = 43 (Total)

KEA-1010 exhibited sedative potency comparable with that of ketamine, yet with more rapid offset in all models investigated. ED_50_ for loss of righting reflex (LORR) following 30 s bolus injection was: KEA-1010 11.4 mg/kg (95% CI 10.6 to 12.3); ketamine 9.6 mg/kg (95% CI 8.5–10.9) (Fig. 3a), with slope of LORR duration vs. dose curves significantly flatter for KEA-1010 than ketamine; KEA 1010 17.0 s/mg/kg (95% CI 12.6 to 21.4); ketamine 69.8 s/mg/kg (95% CI 45.1 to 94.5). (Fig. 3b).

Similarly, KEA-1010 administration resulted in hypnosis (i.e. abolished righting reflex) following 15 s bolus injection at dose 11 mg/kg, and 20 mg/kg rapid infusion over 60 s in all animals undergoing tail flick assessment for antinociceptive action following hypnotic dosing. Duration of righting reflex loss was likewise three-to-four fold lesser in KEA-1010 treated animals compared with ketamine, irrespective of duration of administration. Duration of loss of righting was KEA-1010 115.6 (8.3) vs. ketamine 491.8 (67.7) seconds, *P* < 0.01 following bolus injection; and KEA-1010 72.8 (19.1) vs. ketamine 418.0 (86.4) seconds, *P* < 0.01 following infusion (Fig. 3c, d).

Injection of KEA-1010 and ketamine at 12 mg/kg over 30 s in rats implanted with osmotic mini-pumps (containing either saline *or* morphine) rapidly induced LORR in all animals on days one and six. Duration of LORR was significantly lesser for KEA-1010-treated animals than ketamine-treated animals on both days irrespective of pump content. LORR duration was saline/KEA-1010 54.0 (9.5) vs. saline/ketamine 189.2 (23.8) seconds, *P* < 0.01; and morphine/KEA-1010 90.5 (22.7) vs. morphine/ketamine 247.0 (28.4) seconds, *P* < 0.01 on day 1 (Fig. 3e). LORR duration was saline/KEA-1010 98.8 (17.8) vs. saline/ketamine 277.0 (16.9) seconds, *P* < 0.01; and morphine/KEA-1010 121.2 (13.2) vs. morphine/ketamine 272.5 (12.9) seconds, *P* < 0.01 on day 6 (Fig. 3f). Sedative duration was comparatively greater on day six than day one for KEA-1010 and ketamine treated rats receiving saline via mini-pump (*P* = 0.026 & 0.015 respectively). No difference was observed over time for animals receiving morphine via mini-pump (*P* = 0.18 KEA-1010; *P* = 0.18 Ketamine).

### KEA-1010 attenuates thermal and mechanical nociceptive behaviours

**Fig. 4:**
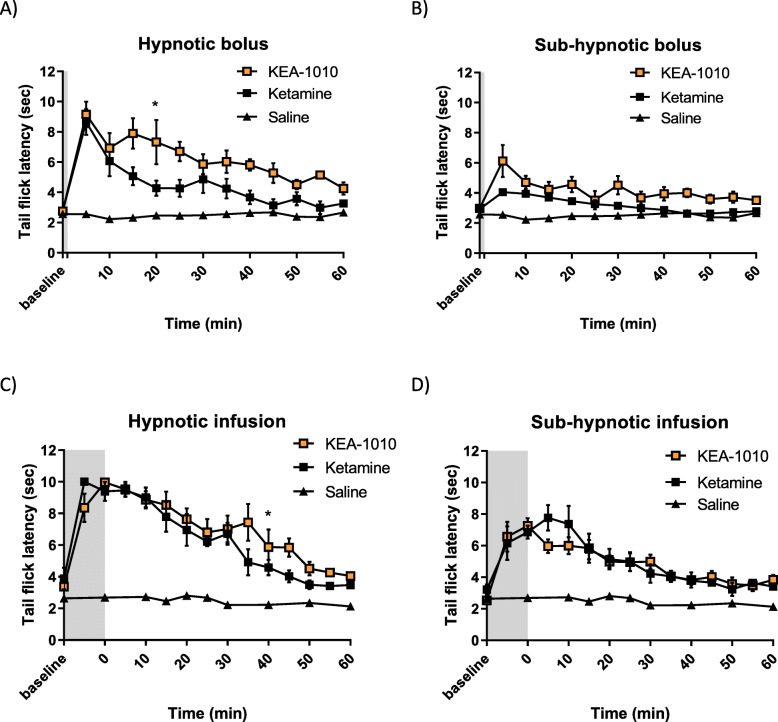
KEA-1010 attenuates thermal nocifensive behaviour. Tail flick latency testing following hypnotic bolus [KEA-1010 11 mg/kg; ketamine 10 mg/kg] (**a**), sub-hypnotic bolus [KEA-1010 5.5 mg/kg; ketamine 5 mg/kg] (**b**), 10 minute hypnotic infusion [KEA-1010 56 mg/kg; ketamine 28 mg/kg] (**c**), and 10-minute sub-hypnotic infusion [KEA-1010 40 mg/kg; ketamine 20 mg/kg] (**d**) in rats. Shaded area denotes duration of drug administration. Data: mean (SEM). *P < 0.05 (KEA-1010 vs. Ketamine; Bonferroni). *N* = 5 all groups

Nociceptive responses to acute thermal and mechanical insults were attenuated by KEA-1010 in a similar fashion to that of the parent compound ketamine in all models investigated. Administration of KEA-1010 and ketamine as pre-emptive analgesia prolonged thermal tail flick latency following hypnotic and sub-hypnotic dosing regimens in rats in dose dependent fashion (Fig. 4a-d). Greater analgesia was observed with KEA-1010 following hypnotic (*P* = 0.0048, *F* = 14.92; RMANOVA) and sub-hypnotic (*P* = 0.026, *F* = 7.45; RMANOVA) bolus injection when compared with ketamine, but no difference following hypnotic (*P* = 0.123, *F* = 2.97; RMANOVA) nor sub-hypnotic (*P* = 0.925, *F* = 0.01; RMANOVA) infusion.

**Fig. 5:**
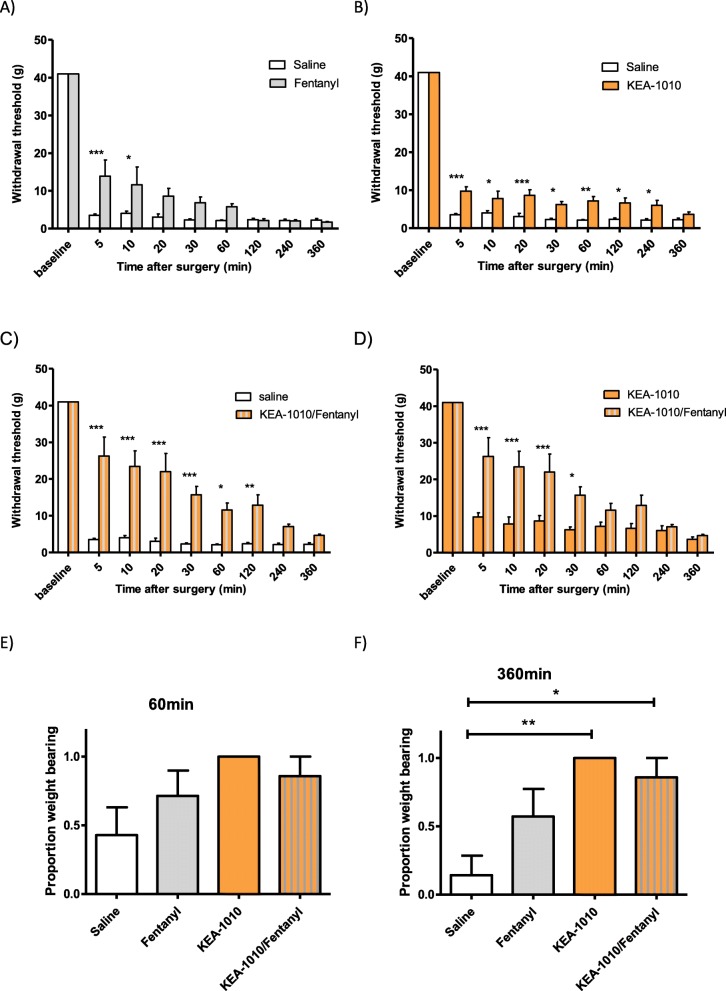
KEA-1010 attenuates post incisional mechanical allodynia; synergy with fentanyl. Pedal withdrawal threshold following hindpaw incision and suture under isoflurane anaesthesia augmented with fentanyl 10 μg/kg (**a**), KEA-1010 20 mg/kg (**b**), or fentanyl 10 μg/kg and KEA-1010 20 mg/kg in rats (**c** & **d**). Weight bearing status at 60 min (**e**), and 360 min (**f**) following surgery. Data mean (SEM). *P < 0.05, **p < 0.01, ***P < 0.001 (Bonferroni). *N* = 7 all groups

Pedal withdrawal thresholds to von Frey filament application following paw incision were demonstrated to be greater in KEA-1010 treated animals when compared with placebo, and comparable to fentanyl in the first post-operative hour (Fig. 5a-d). Persistence of attenuated paw withdrawal to 4 hours post KEA-1010 administration was subsequently observed. Spontaneous weight bearing on the ipsilateral hind-paw was observed to be greater in KEA-1010 treated animals at 6 hours, but not 1 hour (Fig. 5e, f), following injection.

### Synergy with opioid analgesics

KEA-1010 and ketamine injection both augmented morphine induced antinociceptive behaviours in thermal tail flick; and KEA-1010 when co-administered with fentanyl further heightened post-operative mechanical paw withdrawal thresholds.

**Fig. 6:**
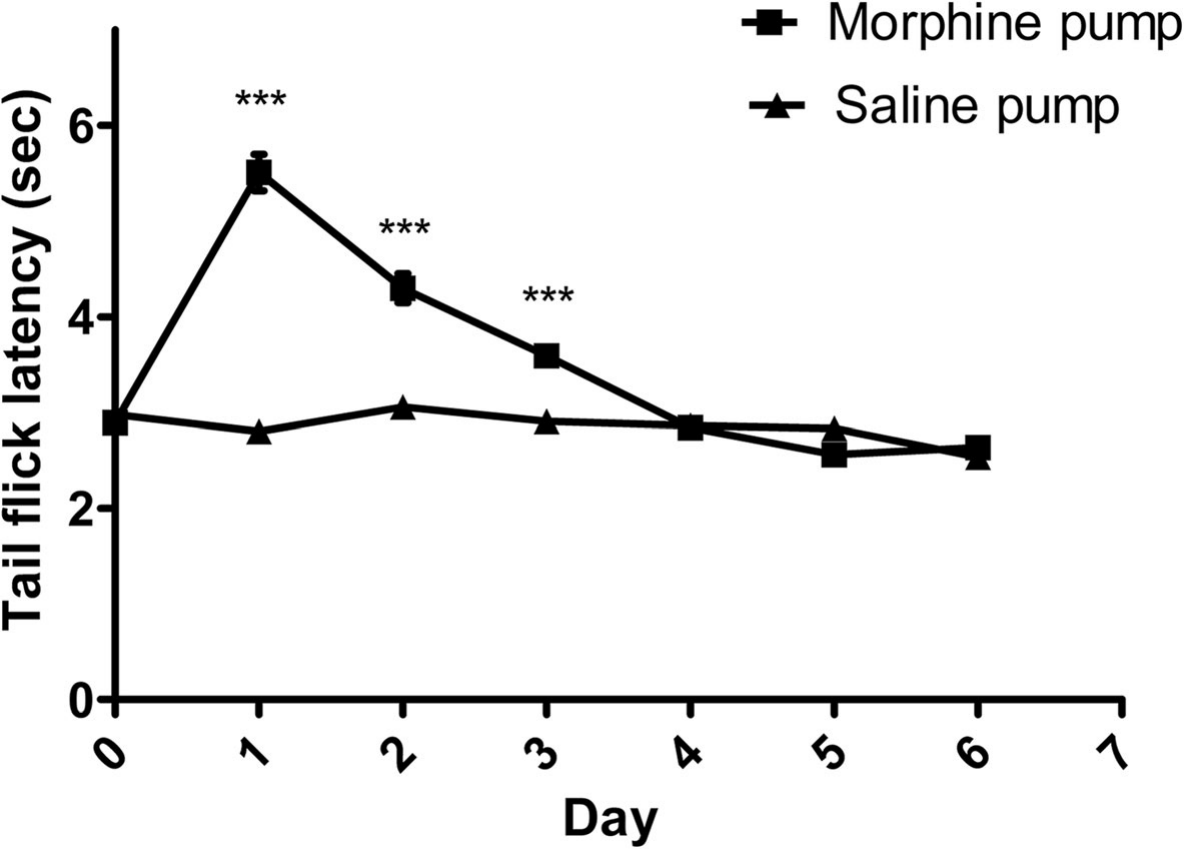
Tolerance to morphine analgesia in rat tail flick latency. Development of tolerance to thermal tail flick stimuli following continuous subcutaneous pump administration of morphine (6 mg/day) in rats take 3–4 days. Data mean (SEM). ***P < 0.001 (Bonferroni). *N* = 18 all groups

**Fig. 7:**
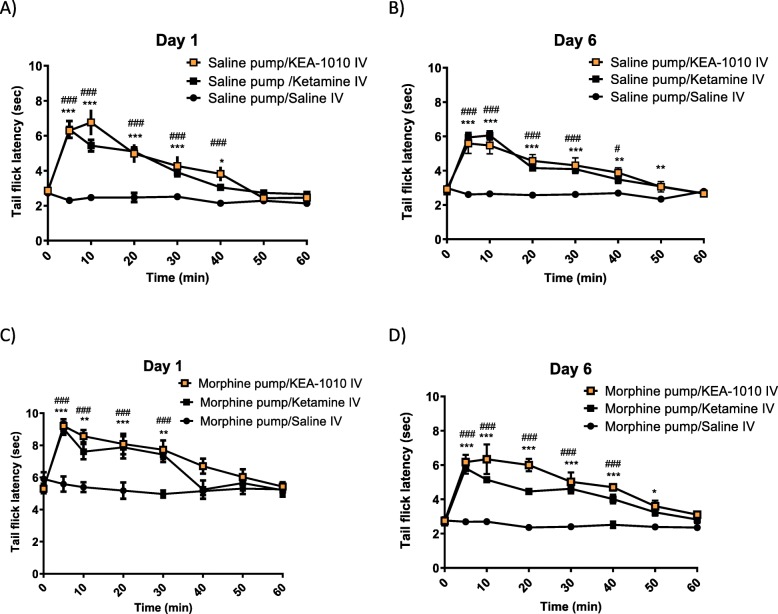
Effectiveness of KEA-1010-induced thermal antinociception in morphine treated rats. Injection of KEA 1010 and ketamine prolonged tail flick latency on day one (**a**) and day six (**b**) in saline minipump rats. KEA-1010 augmented opioid induced analgesic responses in rats receiving morphine via minipump on day one (**c**). KEA remained effective in tail flick prolongation following development of opioid tolerance on day six (**d**). Data mean (SEM). #*P* < 0.05, ##*P* < 0.01, ###*P* < 0.001 (KEA-1010 vs. saline); *P < 0.05, **P < 0.01, ***P < 0.001 (ketamine vs. saline; Bonferroni). *N* = 6 all groups

Rats receiving morphine via osmotic minipump exhibited prolongation of TFL maximal on post-operative day one, and diminishing thereafter in keeping with establishment of opioid tolerance (Fig. 6). Prolongation of thermal tail flick was observed following KEA-1010 and ketamine injection in opioid-naïve rats on day one post saline minipump insertion (KEA-1010 vs. ketamine *P* = 0.478, *F* = 0.54; RMANOVA: Fig. 7a), with KEA-1010 injection on day six eliciting un-diminished TFL prolongation (KEA-1010 vs. ketamine *P* = 0.929, *F* = 0.01; RMANOVA: Fig. 7b). Injection of KEA-1010 and ketamine in morphine minipump animals on day one augmented morphine mediated analgesia with further lengthening of TFL (KEA-1010 vs. ketamine *P* = 0.282, *F* = 1.29; RMANOVA: Fig. 7c). The magnitude and time course of observed effect mirrored that of opioid naïve animals (saline minipiump (Fig. 7a, b) and opioid tolerant rats (Fig. 7d)) and those of non-implanted animals undergoing TFL assessment in 3.5 above.

We further explored the antinociceptive action of KEA-1010 when co-administered with an opioid analgesic in the rat paw incision model. When KEA-1010 and fentanyl were combined as intra-operative analgesia, significant increases in paw withdrawal thresholds to von Frey filament application was observed (Fig. 5c, d). Combination therapy proved superior to either agent alone, with KEA-1010/fentanyl analgesia surpassing that of KEA-1010 alone for 30 min (Fig. 5d). KEA-1010/fentanyl receiving animals exhibited greater spontaneous weight bearing on the operative paw at 6 h, however, no statistically significant difference in spontaneous weight bearing status was observed at 1 hour (Fig. 5e, f).

### Efficacy in opioid tolerance

Following 24 h of continuous morphine treatment, significant antinociception was observed in thermal tail flick latency assessment compared with vehicle treated animals (Fig. 6). On day six, antinociceptive tolerance to morphine was well established, but no morphine-induced hyperalgesia was observed. Administration of KEA-1010 to these opioid tolerant rats resulted in near identical prolongation of tail flick latency to those of opioid naïve animals (KEA-1010 vs. ketamine *P* = 0.075, *F* = 3.95; RMANOVA: Fig. 7d). Likewise, injection of ketamine resulted in TFL prolongation of similar magnitude to that of saline mini-pump subjects.

#### Absence of behavioural aberration

**Fig. 8:**
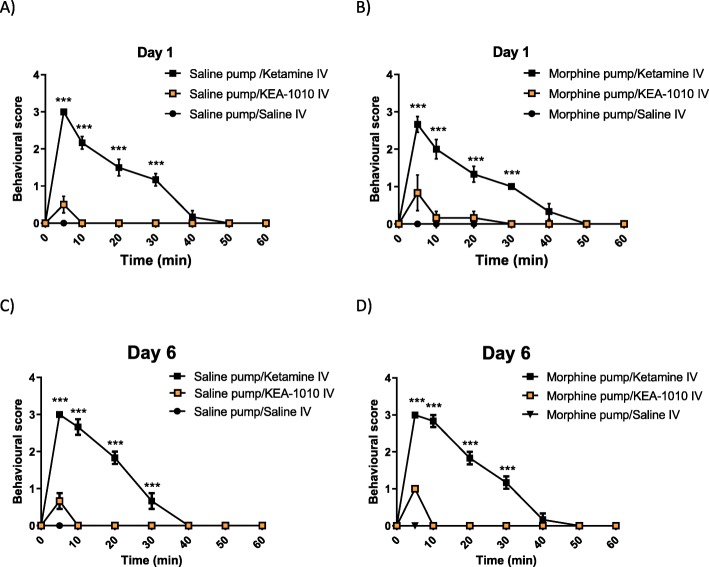
Minimal psychoactivity of KEA-1010 when administered to rats receiving saline or morphine via minipump. Behavioural dysfunction scoring in rats receiving saline and morphine via subcutaneous minipump on days one (**a**, **b**) and day six (**c**, **d**) following injection of KEA-1010 and ketamine. Data mean (SEM). *P < 0.05, **P < 0.01, ***P < 0.001 (KEA-1010 vs. ketamine; Bonferroni). *N* = 6 all groups

Ketamine has been demonstrated to exhibit a myriad of psychoactive effects both acute e.g. dissociative anaesthesia at high dose, and psychotomimesis both on emergence from anaesthesia and at sub-hypnotic dosing. In previous reports KEA-1010 like compounds have been shown to produce significantly less behavioural aberration that the parent compound, ketamine [24, 25]. In conjunction with analgesic assessment of KEA-1010/ketamine injection following mini-pump insertion, we additionally recorded observable parameters denoting behavioural dysfunction. Irrespective of opioid status (naïve, treated, tolerant), rats receiving ketamine exhibited profound behavioural aberration characterised by locomotor activation, ataxic, rearing, and signature stereotaxic head weaving. When quantified in the behavioural dysfunction score ketamine-treated rats demonstrated maximal dysfunction at 5 minutes, with gradual return to normal behavioural patterns over 40 to 50 min. Conversely, behavioural dysfunction scores post KEA-1010 injection were very low, and the duration of mild dysfunction very brief (Fig. 8a-d).

#### Maintained pre-pulse inhibition

**Fig. 9:**
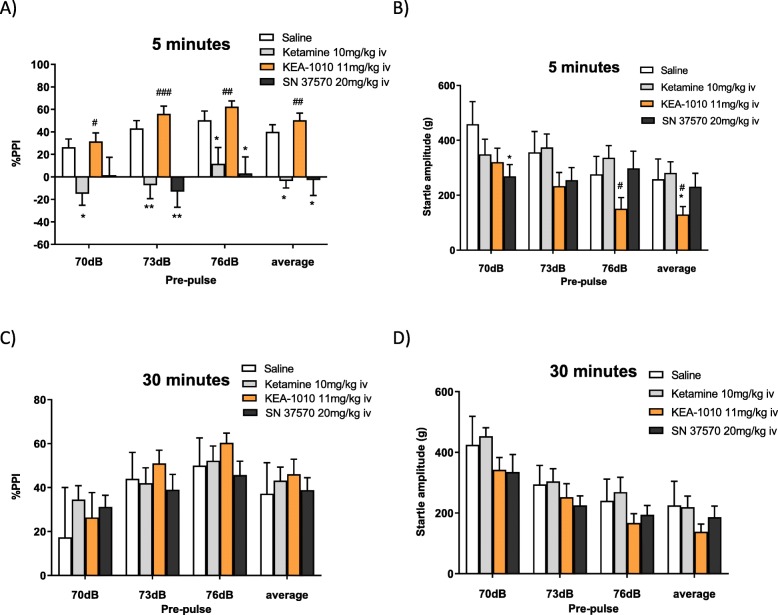
Pre-pulse inhibition is disordered in ketamine treated rats; retained in KEA-1010. Ketamine and KEA-1037, but not KEA-1010, induced pre-pulse inhibition at 5 minutes following drug administration (**a**), amplitude (**b**). No difference in percent pre-pulse inhibition was observed at 30 min following drug administration (**c**), amplitude (**d**). Data mean (SEM). *p < 0.05 (test vs. saline); ** *p* < 0.01 (test vs. saline); #*p* < 0.05 (test vs. ketamine); ## *p* < 0.01 test vs. ketamine; Students T test); ### *p* < 0.001 (test vs. ketamine; Students T test). *N* = 8 all groups

The effect of ketamine, KEA-1010 and KEA-1037 on percent pre-pulse inhibition (PPI) and startle amplitude at 5 minutes are shown in Fig. 9a and b respectively. Significant differences were demonstrated between KEA-1010 and ketamine at all frequencies. In keeping with prior observations of increased psychomimesis, KEA-1037 exhibited a similar PPI disruption fingerprint to that of ketamine. No statistically significant differences were observed in percent PPI or startle amplitude at 30 min after drug administration (Fig. 9c and d).

Ketamine- and KEA-1037-treated animals displayed greater ataxia at 23 and 48 min post dosing compared with vehicle. In contrast, KEA-1010 treated animals displayed significantly less ataxia at 23 min. No further differences were observed between control and drug treated individuals across all metrics at the time-points examined.

## Discussion

Ketamine is a multi-faceted psychoactive drug with clinical applications ranging from traditional dissociative anaesthesia, to analgesia and, more latterly, acute antidepression [23]. In the Emergency room and acute operating theatre the fear of ketamine-induced psychotomimesis has nevertheless curtailed its more widespread application as an analgesic. In the present work we have demonstrated KEA-1010, an *N*-Aliphatic ester-analogue of ketamine, to induce a level of analgesia comparable to that seen with ketamine and opioids, but with considerably lesser adverse effects. KEA-1010 was shown to prolong rat tail flick latency similar to that seen after hypnotic doses of ketamine, but without the ketamine associated sedation and behavioural disturbances (as measured by behavioural dysfunction scoring, and also by pre-pulse inhibition). KEA-1010-mediated antinociception was preserved in morphine-tolerant animals, which suggests a lack of opioid receptor mediation. Furthermore, we showed that – following paw incision – KEA-1010 demonstrated marked analgesic actions in the early post-operative period; manifest both by reduced paw withdrawal to Von Frey hairs, and increased weight bearing. These effects were additive with co-administered fentanyl.

KEA-1010 was designed to be rapidly degraded by tissue carboxylesterases, in order to more rapidly enable recovery from anaesthesia (compared with ketamine) and thereby drastically reduce the time period for the expression of any psychotomimetic adverse effects. An assessment of metabolic stability in recombinantly-expressed carboxylesterase (CES) supersomes confirmed that CES1 type esterases do indeed rapidly degrade the compound to its daughter carboxylic acid, as designed. The same inactive acid metabolite (KEA-1025) has been observed to account for > 95% of all metabolites observed from mouse, rat, dog and human hepatocyte incubation experiments (data not shown).

Bolus and infusion regimens of KEA-1010 induced hypnosis characterised by sustained loss of righting reflex and blunted responses to external stimuli that appeared morphologically identical to that of ketamine. The requirement of higher infusion rates of KEA-1010 to maintain sedation, and rapid return of righting reflex following both bolus and infusion administration, reflects the ultra-rapid degradation by peripheral carboxylesterases (predominantly by the CES1 isoform) in keeping with the known pharmacokinetic profile of these compounds [24, 25, 27]. Modification of known drugs by addition of rapidly-degraded ester moieties has been previously employed to create a suite of ‘soft designer drugs’ (e.g. remifentanil [32], remimazolam [33], and MOC-etomidate [34]). These agents, however, differ from the parent molecule largely in pharmacokinetic profile alone – with rapid elimination enabling ready titration of dose to effect. In all cases the molecular targets (e.g. the μ-opioid receptor in the case of the most clinically recognisable agent, remifentanil) are solitary, and clinical effects largely mimic that of the parent molecule – albeit briefly. (The etomidate analogue MOC-etomidate is also unique for its purported absence of adrenal suppression [34]). In contrast, the observed disparity in time courses of hypnosis and antinociception of KEA-1010 in this study strongly suggests differential activation mechanisms or sites responsible for sedation and analgesia. It is likely that once KEA-1010 enters the brain, it is less readily metabolised (the efficacy of brain CES2 enzymes to metabolise KEA-1010 is far less than peripheral CES1 enzymes). This may lead to accumulation in sites important for analgesia for longer than the compound is seen in the circulation. However, the exact mechanisms and sites responsible for the production of these phenomomen have not yet been elucidated. Further understanding of this drug may lead to greater insights into the different mechanisms of ketamine-induced anaesthesia and analgesia.

KEA-1010 administration in thermal tail flick and mechanical paw withdrawal models induced antinociceptive effects that were both additive with standard opioid medications, and retained in opioid tolerant animals. The magnitude of analgesic effect reported suggests clinical utility with effect size comparable with commonly employed doses of IV opioids in rodent models [35]. Significantly, the analgesic effects of KEA-1010 observed in the opioid tolerance study were seen in the absence of any behavioural aberration as seen in ketamine treated rats. The lack of behavioural disruption and psychotomimetic effects was a key aim in the development of KEA-1010 as a potential opioid-sparing sedative analgesic agent with advantages over ketamine. Rapid enzymatic degradation of the parent compound has been confirmed, as designed for this purpose. However, plasma half-life is of course not the only factor determining the potential for psychotomimetic side effects. Ketamine is a potent NMDAR antagonist, and it is via this channel block that it is thought to induce psychosis in patients. KEA-1010 and its metabolite were analysed for their activity at NMDA channels, and shown to be very weak at this glutamate site compared with ketamine. Thus, KEA-1010 would appear to offer both a short half-life in plasma, limiting the window for the expression of psychosis, and avoid direct block of the psychosis-inducing NMDAR channel. To directly assess and confirm a lack of psychotomimetic effects, KEA-1010 was tested in a rat pre-pulse inhibition study, alongside ketamine and also a structurally-similar analogue from the same development program, KEA-1037 as a positive control. In contrast to KEA-1010 which showed weak NMDA affinity (IC_50_ >  100 μM), KEA-1037 is a potent NMDA blocker (IC_50_ 2.7 μM).

In the PPI study both ketamine and KEA-1037 disrupted the PPI effect in rats, whereas KEA-1010, in contrast, did not disrupt PPI. The behavioural paradigm of pre-pulse inhibition has demonstrated relevance to the sensory disturbances induced by dissociative drugs and serves as an operational measure of sensorimotor gating or filtering [36–38]. Psychotomimetic NMDA receptor antagonists are known to disrupt pre-pulse inhibition in rats [39] with the hallucinogenic effects of PCP- and ketamine-like drugs thought to result, at least in part, from a loss of cortical filtering mechanisms resulting in cortical sensory overload [40]. Our results indicating preserved pre-pulse inhibition for KEA-1010 further supports the assertion that eventual clinical KEA-1010 application might prove devoid of the psychotomimetic side effects seen with ketamine.

Despite more than 50 years of clinical exposure and extensive scientific investigation, the fundamental mechanisms of ketamine’s psychoactivity remain to be fully understood [41]. While non-competitive antagonism at the GluN2B subunit of the NMDA receptor [41, 42] in conjunction with hyperpolarization-activated cyclic nucleotide-gated channel subunit 1 (HCN1) blockade [41–43] are partially thought to underpin ketamine’s sedative effect, multiple additional interactions have been postulated in production of its antinociceptive fingerprint including: dorsal horn sodium channel blockade [42], direct effects on delta opioid receptors and augmentation of mu-receptor function [42, 44], activation of endogenous anti-nociceptive systems through aminergic (serotonergic and noradrenergic) activation and inhibition or reuptake [45], and direct inhibition of nitric oxide synthase [46]. As yet incompletely elucidated intracellular binding sites and modulation of cell chain signalling cascades provide for additional potential mechanisms of analgesic action [47]. Brief screening of potential protein/receptor targets has not supported these as significant mechanisms for the analgesic effect of KEA-1010.

Mechanisms underpinning the activity of KEA-1010, and similar ketamine ester-analogues, likewise remain unclear at present. Previous work has demonstrated a lack of correlation between NMDA receptor affinity and observed anaesthetic/antinociceptive activity for these novel compounds [27]. The lack of significant binding affinity at the NMDA receptor site certainly suggests against this as a primary direct mechanism for KEA-1010s reported beneficial effects. Conversely, NMDA antagonism has been proposed as causative in ketamine’s induction of unwanted psychotogenic side effects [48], a finding in keeping with our present observations of minimal behavioural disruption following KEA-1010 administration. This intriguing finding is the subject of ongoing study but presumably might relate to drug trapping in analgesic brain sites, or the induction of secondary neuronal changes – such as altered gene expression or protein phosphorylation. Previous work has shown that the major metabolites of the drug do not have any analgesic effects to explain this phenomenon.

Absence of significant observable interaction in an NMDAR in vitro assay, however, fails to exclude NMDA related machinery this as a potentially significant drug target. NMDA receptor heterogeneity is widely recognised with varying affinities reported in different brain regions [49]. Furthermore, downstream targets for ketamine (and metabolites) are increasingly seen as pivitol [50]. Analysis of brain transcriptional changes in response to analgesic ketamine analogues has revealed profound large-scale changes in early gene expression in the basolateral amygdala and paraventricular nucleus of the thalamus – in excess to 10 times that of ketamine [51]. In this report we demonstrated significant enrichment was observed in gene pathways related to the function and structure of glutamatergic synapses in respect to: neurotransmitter release, configuration of postsynaptic AMPA receptors, and underlying cytoskeletal scaffolding and alignment following application of KEA-1010 like drugs. The exact nature of these interactions nevertheless remains unclear and are the subject of ongoing study.

The application of animal pain model results to human pain is not straightforward [52]; but we found results that were: broadly consistent with previous opioid and ketamine work; consistent between multiple different animals and experiments; and between both thermal and mechanical stimuli. The von Frey stimulation is thought to be a good test of post-operative mechanical allodynia and hyperalgesia, and so is the closest measure of the clinical analgesic efficacy that might be expected when the drug is given to patients after surgery. It is also considered a good test of drug effects in chronic pain syndromes. Whilst the test clearly does activate mechanoceptors in addition to nociceptors, this is probably an advantage in the translation to the clinical setting, where pain on movement is usually more problematic than pain at rest. The tail-flick thermal analgesia model is a standard model that shows consistent results with opioids.

## Conclusion

KEA-1010 showed substantial analgesic efficacy to both thermal and mechanical nociceptive stimuli in both opioid tolerant and opioid naïve rats. The psychotomimetic signature of the parent compound, ketamine, is largely absent for KEA-1010. These results endorse further development to evaluate its efficacy in human trials.

## Data Availability

The datasets used and/or analysed during the current study are available from the corresponding author on reasonable request.

## Abbreviations

ANOVA: Analysis of Variance
CES: Carboxylesterase
CI: Confidence Interval
dB: Decibel
ED_50_: Effective Dose_50_
G: Grams
IC_50_: Half Maximal Inhibitory Concentration
LORR: Loss of Righting Reflex
Min: Minutes
NMDA: N-methyl-D-aspartate
NMDAR: N-methyl-D-aspartate receptor
PPI: Pre-Pulse Inhibition
RM: Repeated Measures
Sec: Seconds
SEM: Standard Error Mean
TFL: Tail Flick Latency
